# Biopharmaceutical Characteristics of Nifurtimox Tablets for Age‐ and Body Weight‐Adjusted Dosing in Patients With Chagas Disease

**DOI:** 10.1002/cpdd.871

**Published:** 2020-10-08

**Authors:** Heino Stass, Ethel Feleder, Facundo Garcia‐Bournissen, Johannes Nagelschmitz, Boris Weimann, Gustavo Yerino, Jaime Altcheh

**Affiliations:** ^1^ Bayer AG Wuppertal Germany; ^2^ FP Clinical Pharma Buenos Aires Argentina; ^3^ Division of Pediatric Clinical Pharmacology Department of Pediatrics Schulich School of Medicine and Dentistry University of Western Ontario London Ontario Canada; ^4^ CONICET‐GCBA Hospital de Niños Ricardo Gutiérrez and Instituto Multidisciplinario de Investigación en Patologías Pediátricas Buenos Aires Argentina; ^5^ Chrestos Concept GmbH and Co. KG Essen Germany

**Keywords:** bioavailability, exposure, fasting, food effect, *Trypanosoma cruzi*, water dispersible

## Abstract

Treatment of Chagas disease with nifurtimox requires age‐ and body weight‐adjusted dosing, resulting in complex dosing instructions. Appropriate formulations are needed for precise and compliant dosing, especially in pediatric patients. We characterized the biopharmaceutical features of a standard nifurtimox 120‐mg tablet and a 30‐mg tablet developed to improve dose accuracy. Two open‐label, randomized crossover studies were conducted in adult outpatients with Chagas disease. One study investigated whether 4 × 30‐mg tablets and 1 × 120‐mg tablet were bioequivalent and whether tablets can be administered as an aqueous slurry without affecting bioavailability. The second study investigated the effect of a high‐calorie/high‐fat diet versus fasting on the absorption of nifurtimox after a single 4 × 30‐mg dose. Interventions were equivalent if the 90% confidence interval (CI) of their least‐squares (LS) mean ratios for both AUC_0‐tlast_ and C_max_ were in the range of 80%‐125%. The 4 × 30‐mg and 1 × 120‐mg tablet doses were bioequivalent (AUC_0‐tlast_: LS mean ratio, 104.7%; 90%CI, 99.1%‐110.7%; C_max_: LS mean ratio, 101.7%; 90%CI, 89.4%‐115.6%; n = 24). Exposure when giving the 4 × 30‐mg dose as a slurry or as tablets was comparable, with an AUC_0‐tlast_ ratio of 93.2% (84.2%‐103.1%; n = 12) and a slightly decreased C_max_ ratio for the slurry of 76.5% (68.8%‐85.1%). Food improved the bioavailability of nifurtimox substantially (AUC_0‐tlast_ ratio_fed/fasted_, 172%; 90%CI, 154%‐192%; C_max_ ratio_fed/fasted_, 168%; 90%CI, 150%‐187%). The data indicate that the 30‐ and 120‐mg tablets are suitable for dosing adult and pediatric patients accurately; nifurtimox should be administered under fed conditions.

Chagas disease is a chronic and potentially life‐threatening disease caused by the parasite *Trypanosoma cruzi*.^1^ The main route of human transmission is contamination of a bite site or of mucous membranes with parasite‐containing feces from carrier insects.^1^ Vector‐borne transmission is generally limited to North, South, and Central America,^1^ whereas transplacental infection is the main route in nonendemic countries.^2^ Since the early 1990s, several South American countries have focused on eradicating the main insect vector of Chagas disease.^3^ Currently, vector transmission is partially controlled, and most of the new cases occur in urban areas through congenital transmission.^4^ A study was conducted in 2008 in 12 776 children aged 1‐5 years in contiguous rural areas in which uninterrupted vector‐control measures had been in place since 1999. The study determined that the seroprevalence rate of Chagas disease in this pediatric population was 0.24% and was attributable to vertical transmission of the parasite.^3^ In comparison, a 2019 study of children in an area in Bolivia in which vector control is inherently problematic determined that seroprevalence was 22% overall and increased dramatically with age owing to persistent vector transmission.^5^

Nifurtimox (Figure 1) is 1 of only 2 antitrypanosomal agents available for the treatment of *Trypanosoma cruzi* infection.^6^ Antiparasitic treatment during the acute phase of the infection is estimated to be 80%‐90% curative, including in early cases of congenital transmission.^1^ The cytotoxicity of nifurtimox in *Trypanosoma cruzi* has been attributed to metabolism by a type I nitroreductase that leads to production of nitrenium ions, which promote DNA strand breaks, and of saturated open‐chain nitriles that can react nonspecifically with a range of cellular components (Figure 1).^7^ The type I enzyme is rarely found in eukaryotes, except for trypanosomes,^8^ and has no catalytic requirement for oxygen. The analogous mechanism in humans involves a type II nitroreductase that, in the presence of oxygen, converts nifurtimox to an intermediate that undergoes futile cycling, regenerating the parent compound and releasing superoxide anions.^7^

**Figure 1:**
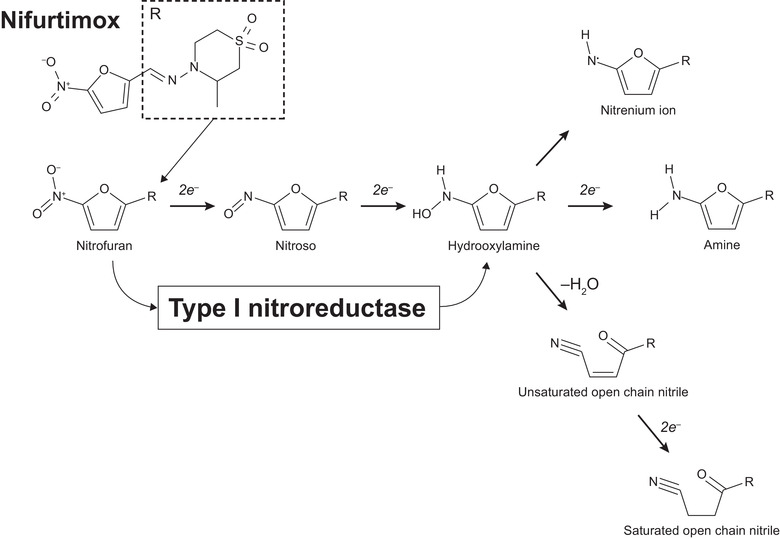
The chemical structure of nifurtimox (CAS No. 23256‐30‐6; molecular weight, 287.29 g/mol) and reduction of the nitrofuran by type I nitroreductase to generate a hydroxylamine via a nitroso intermediate. The hydroxylamine can be metabolized further to form a nitrenium cation (that can promote DNA breakage), the amine form, or unsaturated and then saturated open‐chain nitriles (that could react nonspecifically with a range of cellular components). Reproduced and adapted from Hall et al, *J Biol Chem* 2011^7^).

Pharmacokinetic (PK) studies in animals and in humans indicate that nifurtimox is rapidly absorbed from the gastrointestinal tract, with peak concentrations reached after ≈2 hours.^9^, ^10^ From animal investigations, it has been concluded that concentrations in blood and in the majority of the organs and tissues are similar.^9^ Nifurtimox passes the placental and blood‐brain barriers,^9^ and breastfeeding while taking nifurtimox is not recommended owing to its excretion in breast milk.^11^ However, a population PK model estimate,^11^ subsequently corroborated by a clinical study,^12^ determined that nifurtimox levels in breast milk are typically <10% of the maternal weight‐adjusted dose and below the levels to which infants being treated with nifurtimox would typically be exposed.^11^, ^12^ The drug is rapidly and extensively metabolized,^13^ but the metabolic pathways have not been elucidated. It is known that nitroreductases play an important role in the metabolism of nitrofurans such as nifurtimox,^14^ but detailed characterization of its main metabolites in humans is yet to be reported. Nifurtimox is eliminated with a half‐life of ≈3 hours,^10^ and only 0.5% is excreted unchanged in urine.^13^ However, following oral and intravenous administration of radiolabeled nifurtimox in animals, ≈40% of the radioactive dose was recovered in urine.^9^ To date, there has been no published investigation of biliary and fecal elimination of nifurtimox or of its metabolites in humans.

In terms of the safety profile of nifurtimox, a study of 53 patients with Chagas disease receiving nifurtimox reported anorexia, nausea, headache, amnesia, and weight loss as the most common events.^15^ Another study that compared adverse reactions across different study cohorts receiving nifurtimox determined that digestive disturbances, neurologic toxicity, psychiatric symptoms, and skin hypersensitivity reactions were generally the most common events.^16^ No drug‐drug interactions have been described for nifurtimox, although warnings include the potential for significant adverse side effects with concomitant use of alcohol; studies in rodents indicated that this may be attributable to induction of hepatic P450 reductase, leading to increased nitroreduction of nifurtimox.^17^

The drug is currently supplied as a 120‐mg oral tablet to be taken with food^18^; in children and adolescents the therapeutic dose must be adjusted for age and body weight to account for differences in biodistribution and stages of organ maturation.^6^ The total recommended daily dose of nifurtimox in infants and children aged not greater than 10 years and weighing up to 32 kg is from 10 mg/kg up to a maximum of 20 mg/kg body weight.^18^ In adolescents (aged 11‐16 years) weighing up to 60 kg, the daily dose is 12.5‐15 mg/kg body weight, and in adults (aged ≥17 years) the daily dose is 8‐10 mg/kg. Dosing in adults weighing less than 60 kg should also be in the range of 8‐10 mg/kg, and dosing must be adjusted during treatment if a patient loses weight.^18^ The total daily dose is administered in 3 separate doses (morning, noon, and evening), each after food intake.^18^ Typically the nifurtimox dosing regimen is maintained for at least 60 days.^19^

This relatively complex dosing scheme must be translated into a treatment regimen by individuals often lacking medical training and with limited resources. The existing 120‐mg tablets are scored to facilitate division into two 60‐mg fragments, but further subdivision is commonly necessary, especially for children. Administration of multiple small tablet fragments is prone to inaccurate dosing and to compliance problems among individuals of low or even intermediate body weight. Swallowing tablets can also be difficult for some patients and is not well suited to very young children and infants in whom there is a heightened risk of tablet fragment aspiration.^20^, ^21^

Accuracy of dosing, and thus compliance, could be improved if tablets delivering a dose < 120 mg were available. The availability of different dose strengths of readily divisible tablets may also simplify preparation of each dose, which would be preferable from both administration and compliance perspectives and would yield precise dose increments across all ranges of age and body weight. Tablets that can be dispersed readily as an aqueous slurry address swallowing issues and obviate the need for separate liquid pediatric formulations or administration devices. Accordingly, a divisible 30‐mg nifurtimox tablet was developed to complement the existing divisible 120‐mg tablet. Access to both tablet doses and the option to disperse tablets in water could improve dose accuracy in all age groups and body weights, including dose‐appropriate treatment of children with low body weight. Improving dose accuracy and encouraging compliance are likely to have positive implications for safety and patient outcomes.

The aims of the 2 studies reported here were to compare the bioavailability and PK of a new 30‐mg nifurtimox tablet administered whole or as an aqueous slurry with those of a 120‐mg tablet (study A) and to examine the impact of food on the absorption of these formulations (study B). Both studies also examined the safety and tolerability of nifurtimox.

## Methods

Study protocols and informed consent documentation were approved by an independent ethics committee (Independent Ethics Committee for Clinical Pharmacology Research, Buenos Aires, Argentina). The studies were conducted in accordance with the ethical principles of the Declaration of Helsinki and in compliance with the International Conference on Harmonization Good Clinical Practice guidelines. All participants gave written informed consent before enrollment. The studies were conducted at FP Clinical Pharma SRL, Buenos Aires, Argentina.

### Study Designs

Study A was a phase 1 single‐center, open‐label, randomized, 3‐intervention crossover study designed to assess the bioequivalence of the novel 30‐mg oral tablet and the currently marketed 120‐mg tablet under fed conditions in adult patients with Chagas disease and to characterize the PK of nifurtimox after ingestion as an aqueous slurry. The study was conducted between November 2013 and September 2014 (ClinicalTrials.gov Identifier: NCT01927224).

Study B was a phase 1 single‐center, open‐label, randomized crossover study conducted between December 2015 and August 2016 (ClinicalTrials.gov Identifier: NCT02606864) to investigate the effect of a high‐calorie, high‐fat meal versus a fasting state on the PK of four 30‐mg nifurtimox tablets administered as a single dose to adult patients with Chagas disease. Both studies also examined the safety and tolerability of nifurtimox.

The 2 studies each had an initial 4‐week screening period followed by 2 treatment periods that were separated by a washout of at least 5 days. Participants arrived 12 hours before dosing and remained at the study center for 24 hours after dosing. Doses were swallowed immediately after a high‐fat, high‐calorie breakfast (unless patients were fasting [study B]) with ≈240 mL of water while the participant was in a sitting position. The participant then remained supine for 4 hours postdose; water intake was not permitted for 2 hours postdose. Further meals on treatment days were standardized and provided about 5, 8, and 10 hours postdose, with subjects instructed to eat a normal diet during the washout phase and in the follow‐up period (7‐14 days after administration of the second dose). In both studies, participants were assigned a randomization number after confirmation of eligibility at screening, and these numbers were randomly assigned to 1 of the 2 intervention sequences in their assigned group according to a computer‐generated randomization list.

#### Study A

**Figure 2:**
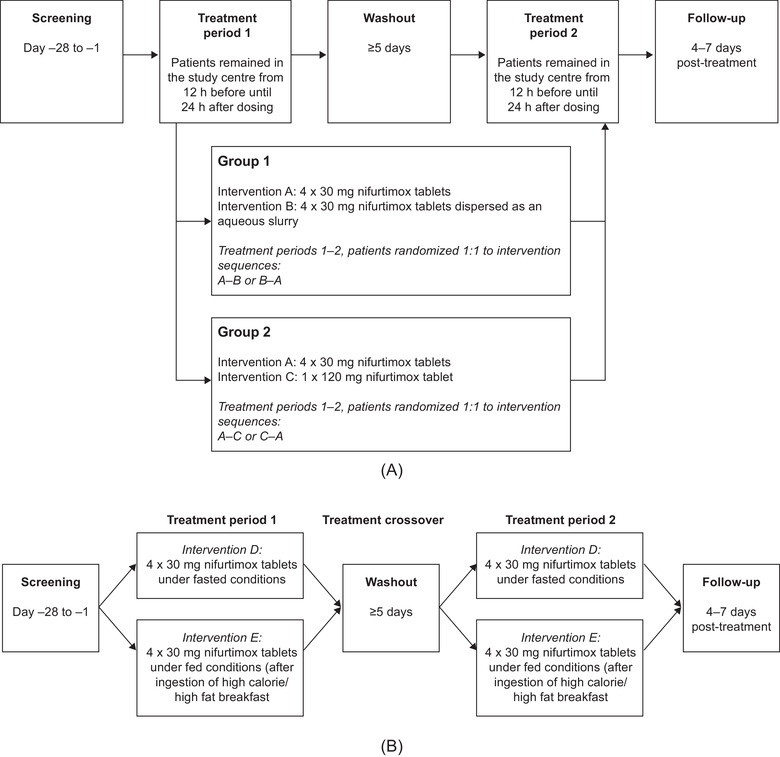
Study designs. (A) In study A, group 1 investigated the pharmacokinetic characteristics of nifurtimox administered as tablets or slurry, and group 2 investigated the bioequivalence of the 30‐ and 120‐mg tablets. Intervention sequences are shown in each group; all dosing was under fed conditions. (B) Study B compared the bioavailability of nifurtimox under fed and fasting conditions.

In each treatment period, a total nifurtimox dose of 120 mg was administered 30 minutes after a standardized high‐fat, high‐calorie breakfast (1051 kcal) that was preceded by a fasting period of at least 10 hours. Three interventions were specified: (A) 4 nifurtimox 30‐mg tablets taken whole with water; (B) 4 nifurtimox 30‐mg tablets dispersed in an aqueous slurry; (C) 1 × nifurtimox 120‐mg tablet taken whole with water. Participants were randomized to 1 of 2 intervention sequences: in group 1 (tablet‐slurry bioavailability), patients were randomized 1:1 to intervention sequences A‐B or B‐A, and in group 2 (tablet bioequivalence) to sequences A‐C or C‐A (Figure 2A).

#### Study B

Two interventions were specified: (D) nifurtimox 120 mg (4 × 30‐mg tablets) following a fasting period of at least 10 hours; (E) nifurtimox 120 mg (4 × 30‐mg tablets) 30 minutes after a standardized high‐fat, high‐calorie breakfast (800‐1000 kcal) preceded by a fasting period of at least 10 hours. Participants were enrolled in 1 of 2 intervention sequences: D‐E or E‐D (Figure 2B).

In both studies, blood and urine samples were taken for safety analysis at baseline, after treatment, and at follow‐up. Blood samples were analyzed for hematology parameters; clotting status; clinical chemistry; hepatitis B, hepatitis C and human immunodeficiency virus seroconversion; and pregnancy. Urinalysis parameters included pH, hemoglobin, urobilinogen, erythrocytes, protein, ketones, bilirubin, glucose, and urine sediment. Blood samples for PK analysis were taken within 1 hour predose and 0.25, 0.5, 0.75, 1, 1.5, 2, 2.5, 3, 4, 6, 8, 10, 12, 16, and 24 hours postdose. All samples were stored at −20°C between collection and analysis and analyzed within 116 days of sampling.

### Study Participants

Studies A and B were conducted in patients in the indeterminate stage of Chagas disease who were otherwise healthy, that is, no cardiac involvement or any other pathology that could affect interpretation of the study results. The diagnosis of chronic Chagas disease was made by 2 positive serological tests for *Trypanosoma cruzi*. Eligible patients were aged 18‐45 years with a body mass index (BMI) of at least 18 and no greater than 29.9 kg/m^2^. Women of childbearing age and sexually active men were required to use 2 methods of contraception during and for 12 weeks after completing participation in a study. Full inclusion and exclusion criteria for both studies are provided in the supplement (Tables S1 and S2).

### Safety and Tolerability Assessment

Adverse events (AEs) were defined as any untoward medical occurrence in a participant after providing written informed consent and were recorded throughout the study until the last follow‐up visit; AEs were summarized using Medical Dictionary for Regulatory Activities terms (version 19.0) and classified as treatment‐emergent AEs (TEAEs) if they first appeared or worsened after the first dose of study drug and up to 30 days after the end of study treatment.

### Nifurtimox Quantitation

Nifurtimox was assayed using liquid chromatography with tandem mass spectrometry detection (inVentiv Health Clinical, Quebec City, Quebec, Canada). Study samples of human plasma containing dipotassium ethylenediaminetetraacetate were thawed at room temperature — 50 μL mixed with 100 μL of the internal standard (nifurtimox‐d_8_) and 500 μLof assay buffer (500 mM 2‐amino‐2‐[hydroxymethyl]‐1,3‐propanediol). Samples underwent liquid‐liquid extraction by shaking with 4 mL of a 90/10 methyl tert‐butyl ether/hexane mixture for 15 minutes followed by centrifugation at 2000*g* for 5 minutes at room temperature. Samples were flash‐frozen in a methanol/dry ice bath, and the organic phase removed to a new tube and evaporated to dryness. Samples were reconstituted in methanol/water (46%/54%) containing 1 mM ammonium formate and 0.1% formic acid (also the chromatography mobile phase) and separated isocratically at room temperature at 1 mL/min using a Life Science ACE 3 C18 reversed‐phase column (4.6 × 30 mm, 3 μm) on an Agilent 1100 liquid chromatography system coupled to an Applied Biosystems/Sciex API 4000 triple quadrupole mass spectrometer in positive TurboIonSpray ionization mode. After ionization at atmospheric pressure the mass transition signals of nifurtimox (288/148 amu; dwell time, 340 milliseconds) and the nifurtimox‐d_8_ internal standard (296/156 amu; dwell time, 160 milliseconds) were recorded using multiple‐reaction monitoring. In study A the interrun accuracy was −0.22% to 0.97% of bias, and the interrun precision was 2.37%‐4.09% of the coefficient of variation (CV) calculated from the quality control samples. In study B, the interrun accuracy was −1.30% to −3.33% of bias, and the inter‐run precision was 3.59%‐5.48% of CV calculated from quality control samples. Quantitation was by the peak‐area ratio method. Weighted (1/x^2^) linear regression was performed to determine the concentrations of the analyte. All peak integrations were generated by Applied Biosystems/Sciex Analyst software version 1.6.1, and all regressions were generated by ThermoElectron Corporation Watson LIMS software, version 7.4.1. The calibration range (lower limit of quantitation [LLOQ] to the upper limit) was 10‐2000 μg/L. Only values above the LLOQ were used to determine PK parameters. For both studies the concentration‐time courses of nifurtimox were prepared separately by intervention. Arithmetic and geometric means, standard deviation (SD), median, and % CV were calculated for each sampling point. Means were only calculated if at least two‐thirds of the individual data were measured and were above the LLOQ.

### PK Calculations

The following primary PK parameters were calculated based on the nifurtimox plasma concentration‐over‐time data: area under the concentration curve from baseline to last measurable concentration (AUC_0‐tlast_), maximum observed concentration (C_max_), and time to reach C_max_ (t_max_ [study B]). Secondary PK parameters included: area under the concentration curve (AUC), C_max_ normalized to dose and body weight (C_max,norm_), AUC from baseline to infinity normalized for dose/kg body weight (AUC_norm_ [study A]), t_max_, half‐life associated with the terminal slope (t_½_), and apparent total body clearance (CL/F). In both studies, PK parameters were calculated based on a model‐independent noncompartmental method using WinNonlin (v.5.3; Pharsight Corporation/Certara, Princeton, New Jersey).

### Statistical Analyses

A sample size of 12 subjects in study A group 1 was considered adequate to compare the 2 modes of drug ingestion (tablet vs aqueous slurry) descriptively and to verify assumptions made about within‐subject variability. In study A group 2, it was calculated that 21 participants would have 90% power to demonstrate the bioequivalence of the 2 interventions (4 × 30‐mg tablets; 1 × 120‐mg tablet) with respect to AUC_0‐tlast_ and C_max_ if, after a single dose of nifurtimox, the ratios of the least‐squares (LS) means of these parameters in the 2 intervention groups were in the range of 95%‐105% (with 90% confidence intervals [CIs] for both parameter ratios in the range of 80%‐125% [specifically, the lower bound was ≥80.00% and the upper bound ≤125.00%, when rounded to 2 decimal places]) and if the within‐subject % CV for these parameters was <18%. Enrollment of 24 individuals was planned in study A group 2 to allow for possible study dropout.

For study B, it was calculated that a sample size of 32 participants would have 82% power (α = 0.05) to rule out a food effect if the fed/fasted ratio for parameters AUC, AUC_0‐tlast_, and C_max_ were all in the range of 95%‐105% (with 90%CI for these ratios in the range of 80%‐125%), and the within‐subject % CV for these parameters was less than reference % CV values determined in study A (AUC_0‐tlast_, 11.19; C_max_, 26.36). Target recruitment of 36 individuals allowed for dropout.

Statistical analysis in both studies was performed using SAS (version 9.2; SAS Institute Inc., Cary, North Carolina). Analysis of bioequivalence in study A was confirmatory; all other analyses were exploratory with no adjustment for type I error. In study A, all individuals with at least 1 valid PK profile were included in the PK analysis set. In study B, the PK analysis set comprised all individuals who completed both treatment periods with a valid set of PK samples. All individuals who received at least 1 dose of study drug were included in the safety set for both studies. Baseline demographic and safety data in both studies were summarized descriptively.

For study A (groups 1 and 2), the AUC_0‐tlast_ and C_max_ of nifurtimox were analyzed assuming log‐normally distributed data, using analysis of variance (ANOVA) including sequence, subject (sequence), period, and treatment effects. Point estimates (LS means) and exploratory 90%CIs for the ratios (group 1: slurry/tablets; group 2: 4 × 30‐mg tablets/1 × 120‐mg tablet) for AUC_0‐tlast_ and C_max_ were calculated by re‐transformation of the logarithmic data using the intraindividual SD of the ANOVA. For study B, AUC, AUC_0‐tlast_, and C_max_ of nifurtimox were analyzed assuming log‐normally distributed data, using ANOVA including sequence, subject (sequence), period, and treatment effects. Point estimates (LS means) and exploratory 90%CIs for the ratios (fed/fasted) for AUC_0‐tlast_ and C_max_ were calculated by retransformation of the logarithmic data using the intraindividual SD of the ANOVA.

## Results

### Study A

#### PK Characteristics of Nifurtimox 120 mg Administered as 4 × 30‐mg Tablets or as an Aqueous Slurry

Study A enrolled 39 individuals, of whom 37 were randomized; 1 randomized participant withdrew before receiving study drug. All 36 individuals who received study drug (group 1, n = 12; group 2, n = 24) completed the study and were included in both the safety and PK analysis sets; all participants in group 1 (12 of 12) and 66.7% in group 2 (16 of 24) were women. Mean age, BMI, and weight were similar in the 2 groups and in the intervention sequences within each group; all participants were white (Table 1).

**Table 1:** Patient Characteristics at Baseline by Study and Intervention Sequence

	Study A								
	Group 1			Group 2			Study B		
Characteristic	A‐B (n = 6)	B‐A (n = 6)	Total (n = 12)	A‐C (n = 12)	C‐A (n = 12)	Total (n = 24)	D‐E (n = 18)	E‐D (n = 18)	Total (n = 36)
Women, n (%)	6 (100)	6 (100)	12 (100)	7 (58.3)	9 (75.0)	16 (66.7)	16 (88.9)	16 (88.9)	32 (88.9)
Age, years^a^	33.3 (22‐42)	34.3 (27‐39)	33.8 (22‐42)	32.5 (18‐44)	32.3 (22‐45)	32.4 (18‐45)	34.3 (27–45)	33.6 (26–43)	33.9 (26–45)
Weight, kg	62.9 ± 4.4	66.8 ± 12.5	64.9 ± 9.2	64.5 ± 14.7	63.3 ± 11.4	63.9 ± 12.9	63.6 ± 10.7	65.3 ± 10.9	64.4 ± 10.7
BMI, kg/m^2^	25.8 ± 2.8	26.3 ± 2.5	26.1 ± 2.5	24.6 ± 3.4	24.2 ± 3.2	24.4 ± 3.2	25.7 ± 3.5	26.9 ± 2.6	26.3 ± 3.1

BMI, body mass index.

Study A: intervention A, 4 × 30‐mg tablets; intervention B, 4 × 30 mg aqueous slurry; intervention C, 1 × 120‐mg tablet (all dosing was under fed conditions). Study B: intervention D, 4 × 30‐mg tablets, fasted; intervention E, 4 × 30‐mg tablets, fed.

a Mean (range); other data are mean ± standard deviation unless stated otherwise.

**Figure 3:**
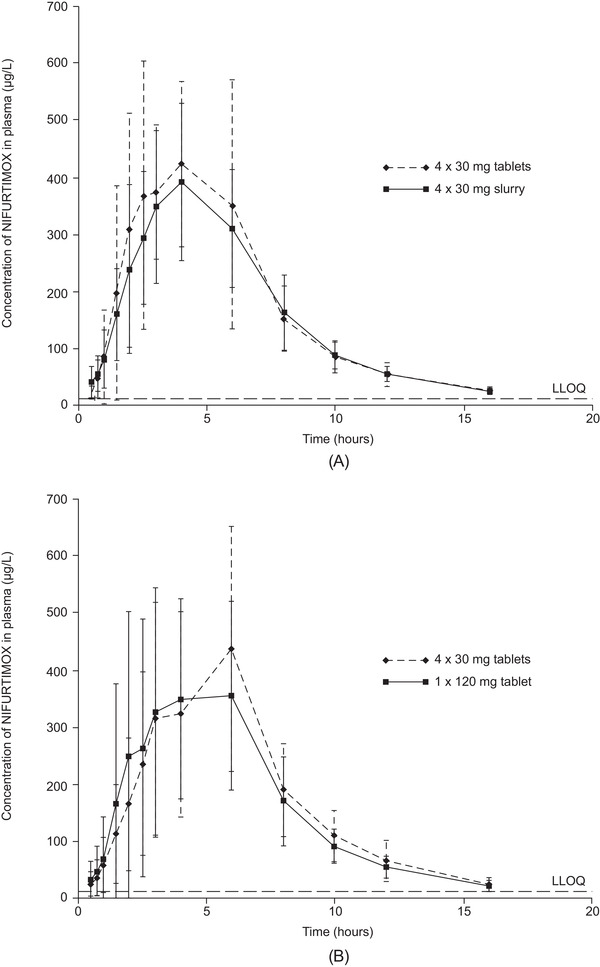
Nifurtimox plasma concentrations for participants in study A. (A) Group 1: 4 × 30‐mg tablets or 4 × 30‐mg aqueous slurry under fed conditions (n = 12), (B) group 2: 4 × 30‐mg tablets or 1 × 120‐mg tablet under fed conditions (n = 24). Data are arithmetic mean and standard deviation. LLOQ, lower limit of quantitation.

The arithmetic mean plasma concentrations of nifurtimox over time in group 1 (receiving 120 mg nifurtimox as 4 × 30‐mg tablets or 4 × 30 mg as an aqueous slurry) are shown in Figure 3A, and PK parameters are summarized in Table 2 (geometric mean plasma concentration of nifurtimox over time in group 1 and geometric mean [% CV] group values for PK parameters are shown in Figure S1A and Table S3, respectively). Administered as an aqueous slurry, nifurtimox had an AUC_0‐tlast_ similar to that of the corresponding tablet formulation. The point estimate (LS mean [90%CI]) ratio of 4 × 30 mg aqueous slurry versus 4 × 30‐mg tablets for AUC_0–tlast_ was 93.2% (84.2%‐103.1%) and for C_max_ was 76.5% (68.8%‐85.1%). The 90%CIs for AUC_0–tlast_ lay within the predefined range for equivalent bioavailability, indicating no difference between the 2 intervention groups in terms of systemic exposure to nifurtimox. When administered as an aqueous slurry, peak exposure to nifurtimox (C_max_) was ≈24% lower than that achieved with the same dose administered as tablets.

**Table 2:** Pharmacokinetic Parameters of Nifurtimox in Study A (Mean ± SD; PK Analysis Set)^a^

	Group 1		Group 2	
Parameter	Intervention A: 4 × 30‐mg Tablets (n = 12)	Intervention B: 4 × 30‐mg Aqueous Slurry (n = 12)	Intervention A: 4 × 30‐mg Tablets (n = 24)	Intervention C: 1 × 120‐mg Tablet (n = 24)
AUC, μg·h/L	2830 ± 542	2680 ± 526	2750 ± 710	2620 ± 607
AUC_0–tlast_, μg·h/L	2720 ± 535	2550 ± 519	2640 ± 693	2520 ± 594
AUC_norm_, μg·h/L	1510 ± 233	1440 ± 315	1420 ± 265	1350 ± 242
C_max_, μg/L	586 ± 158	450 ± 114	552 ± 186	540 ± 178
C_max,norm_, kg/L	309 ± 59.2	240 ± 56.8	287 ± 99.8	282 ± 99.2
t_max_, h^a^	4.0 (2.0‐6.0)	4.0 (2.0‐8.0)	4.0 (2.0‐6.0)	4.0 (2.0‐6.0)
t_½_, h	3.32 ± 0.39	3.88 ± 1.94	2.69 ± 0.56	2.96 ± 0.78

AUC_0‐tlast_, AUC from baseline to last measurable concentration; AUC, area under the plasma concentration curve; AUC_norm_, AUC from baseline to infinity normalized for dose/kg body weight; C_max_, maximum observed concentration; C_max,norm_, C_max_ normalized to dose and body weight; PK, pharmacokinetic; SD, standard deviation; t_½_, half‐life; t_max_, time to reach C_max_. All dosing was under fed conditions.

a Median (range).

#### Equivalence of 4 × 30‐mg and 1 × 120‐mg Nifurtimox Tablets

The arithmetic mean plasma concentrations of nifurtimox over time in group 2 (receiving 4 × 30‐mg tablets or 1 × 120‐mg tablet) are shown in Figure 3B, and PK parameters are summarized in Table 2 (geometric mean plasma concentration of nifurtimox in group 2, and the geometric mean [% CV] group values for PK parameters are shown in Figure S1B and Table S3, respectively). Median t_max_ was 4 hours with both interventions, and the estimated arithmetic mean half‐life in the 2 intervention groups differed by about 16 minutes. For the ratio of 4 × 30‐mg tablets versus 1 × 120‐mg tablet, the point estimate (LS mean [90%CI]) ratio for AUC_0‐tlast_ was 104.7% (99.1%‐110.7%) and for C_max_ was 101.7% (89.4%‐115.6%). Bioequivalence was demonstrated because the 90%CIs of the point estimates for both parameters lay within the predefined range (80%‐125%).

### Study B

#### Comparison of Bioavailability of Nifurtimox Administered as 4 × 30‐mg Tablets Under Fed or Fasting Conditions

Study B enrolled 39 individuals, of whom 36 were randomized (sequence D‐E, n = 18; sequence E‐D, n = 18). One participant randomized to the E‐D (fed‐fasted) intervention sequence discontinued, withdrawing consent after the first dose of study drug. All 36 participants were included in the safety set; the 35 individuals who completed the study were included in the PK set. Participants in both intervention sequences were well matched by sex, age, and weight. Most participants were female (88.9%; n = 36) and Hispanic or Latino (91.7%; n = 33); all were white.

**Figure 4:**
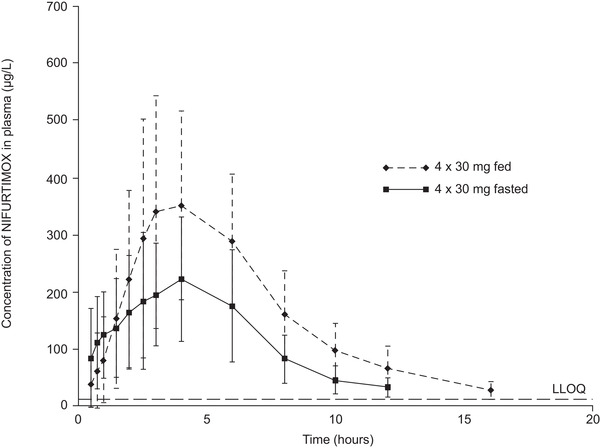
Nifurtimox plasma concentrations for participants in study B receiving 4 × 30‐mg tablets in the fasted or fed states (n = 35). Data are arithmetic mean and standard deviation. LLOQ, lower limit of quantitation.

**Figure 5:**
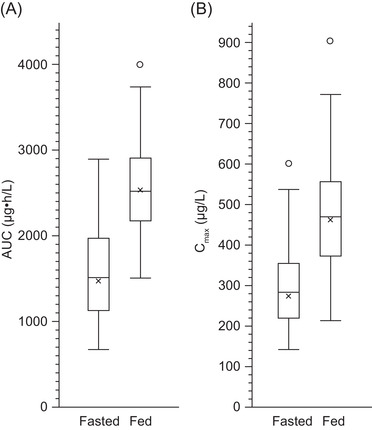
AUC and C_max_ for nifurtimox in plasma under fasting and fed conditions in study B. x, mean; horizontal line, median; box, IQR; error bars, data range to a maximum of 1.5 × IQR; open circle, data outlier. AUC, area under the plasma concentration curve; C_max_, maximum observed concentration; IQR, interquartile range.

The arithmetic mean plasma concentrations of nifurtimox over time in the groups receiving 4 × 30‐mg tablets under fasted (group D) or fed (group E) conditions are shown in Figure 4, and PK parameters are summarized in Table 3 (geometric mean plasma concentration of nifurtimox over time in groups D and E, and the geometric mean [% CV] group values for PK parameters are shown in Figure S2 and Table S4, respectively). Food taken before dosing increased systemic exposure to nifurtimox by at least 71% compared with fasting conditions. The estimated ratios (LS mean [90%CI]) for nifurtimox under fed conditions versus fasted conditions were 171% (154%‐191%) for AUC and 172% (154%‐192%) for AUC_0‐tlast_. Peak exposure increased by a similar degree under fed conditions (C_max_, 168% [150%‐187%]; Figure 5). The 90%CIs of all ratios (fed/fasted) did not lie within the 80%‐125% range, indicating a pronounced food effect. Food intake also reduced the rate of absorption of nifurtimox, increasing median t_max_ by 1 hour (Table 3).

**Table 3:** Pharmacokinetic Parameters of Nifurtimox in Study B (Mean ± SD; PK Analysis Set)^a^

Parameter	Intervention D: 4 × 30‐mg Tablets, Fasted (n = 35)	Intervention E: 4 × 30‐mg Tablets, Fed (n = 35)
AUC, μg·h/L	1590 ± 606	2590 ± 547
AUC_0‐tlast_, μg·h/L	1490 ± 568	2450 ± 519
CL/F, L/h	87.4 ± 35.0	48.4 ± 10.4
C_max_, μg/L	295 ± 109	490 ± 163
t_max_, h^a^	3.0 (0.5‐6.1)	4.0 (1.0‐8.0)
t_½_, h	3.24 ± 1.15	3.24 ± 0.91

AUC_0‐tlast_, AUC from baseline to last measurable concentration; AUC, area under the concentration curve; CL/F, apparent total body clearance; C_max_, maximum observed concentration; PK, pharmacokinetic; SD, standard deviation; t_½_, half‐life; t_max_, time to reach C_max_.

a Median (range).

### Safety

In study A, a total of 8 of the 12 subjects in group 1 (66.7%) and 9 of the 24 subjects in group 2 (37.5%) reported at least 1 TEAE. Of the 36 subjects in study B, 5 (13.9%) reported a TEAE (Table 4). All TEAEs in both studies were considered drug related, except for 1 mild headache (study B). All TEAEs resolved completely, most within 1 day. Three individuals were treated for vomiting and 2 for headache. The TEAEs experienced by subjects in both studies were mild or moderate in intensity, and there were no serious AEs or TEAEs leading to discontinuation of nifurtimox. The most common TEAEs in both studies were known side effects of nifurtimox, including vomiting, nausea, abdominal pain, and headache, and no notable differences in TEAEs between intervention groups in either study were observed (Table 4). No procedure‐related AEs were reported in either study, there were no notable differences in abnormal laboratory test results between formulations in either study, and there were no clinically relevant changes in any laboratory parameter.

**Table 4:** Treatment‐Emergent Adverse Events by Study and Intervention Sequence (Safety Analysis Set)

	Study A								
	Group 1			Group 2			Study B		
Event	Intervention A: 4 × 30‐mg Tablets (n = 12)	Intervention B: 4 × 30 mg Aqueous Slurry (n = 12)	Total^a^ (n = 12)	Intervention A: 4 × 30‐mg Tablets (n = 24)	Intervention C: 1 × 120‐mg Tablet (n = 24)	Total^a^ (n = 24)	Intervention D: 4 × 30‐mg Tablets, Fasted (n = 35)	Intervention E: 4 × 30‐mg Tablets, Fed (n = 36)	Total^a^ (n = 36)
Any TEAE	7 (58.3)	4 (33.3)	8 (66.7)	5 (20.8)	5 (20.8)	9 (37.5)	3 (8.6)	2 (5.6)	5 (13.9)
Drug‐related TEAE	7 (58.3)	4 (33.3)	8 (66.7)	5 (20.8)	5 (20.8)	9 (37.5)	3 (8.6)	1 (2.8)	4 (11.1)
Most common TEAEs									
Abdominal pain	0	0	0	1 (4.2)	1 (4.2)	2 (8.3)	1 (2.9)	0	1 (2.8)
Nausea	0	2 (16.7)	2 (16.7)	1 (4.2)	4 (16.7)	4 (16.7)	1 (2.9)	1 (2.8)	2 (5.6)
Vomiting	4 (33.3)	3 (25.0)	6 (50.0)	0	1 (4.2)	1 (4.2)	0	1 (2.8)	1 (2.8)
Headache	4 (33.3)	3 (25.0)	5 (41.7)	3 (12.5)	1 (4.2)	4 (16.7)	2 (5.7)	1 (2.8)	3 (8.3)

TEAE, treatment‐emergent adverse event.

Data are n (%).

a Total number of individuals over both treatment periods.

## Discussion

We conducted 2 studies comparing the PK of a new nifurtimox 30‐mg tablet that can be administered as an aqueous slurry with that of the marketed 120‐mg tablet, and also examined the impact of food on nifurtimox absorption. The studies demonstrated that when taken after food, the new nifurtimox 30‐mg tablet formulation administered at a total dose of 120 mg is bioequivalent to the standard 120‐mg tablet both in terms of systemic exposure and bioavailability. The 30‐mg tablet is scored for ease of division, so the availability of both dose levels will permit doses to be adjusted in relation to age and body weight in increments of 15, 30, 60, and 120 mg. Furthermore, the option to administer the drug as an aqueous slurry will facilitate treatment of very young patients and individuals of any age who find it difficult to swallow tablets.

In the context of the current clinical development program for pediatric nifurtimox, target daily dosing is 8‐10 mg/kg in adolescents weighing >40 kg, and 10‐20 mg/kg in children younger than 10 years of age weighing < 40 kg. The total daily dose is typically divided into 3 doses administered every 8 hours.^18^ Dose adjustment according to body weight is frequently unwieldy and inaccurate when a 120‐mg tablet is the only dose available. Administration of quarter tablets is the only option when trying to optimize dose without exceeding recommended levels, and may need to be readjusted if patients lose weight while on treatment.^18^ Moreover, dosing may become unsafe when the accuracy of dose adjustment in young children or infants depends on dividing a tablet with precision. Dose adjustment will be greatly facilitated when 15‐mg dose increments can be deployed.

The option to disperse nifurtimox tablets in an aqueous slurry also facilitates dose adjustment, and confers a useful safety advantage, avoiding possible bronchial obstruction in infants following unintentional aspiration of a tablet. Foreign‐body aspirations occur mainly in children but are also reported among the elderly, and it has been estimated that 7% of all such aspirations are medicinal pills.^20^, ^21^ Accordingly, administration of medicinal pills should be avoided in high‐risk patients such as infants and those with swallowing disorders.^21^ When dispersed as an aqueous slurry and administered to individuals after food, the concentration of nifurtimox in plasma emulated that achieved by administering four 30‐mg tablets, and overall systemic exposure to nifurtimox was essentially the same for both modes of administration.

The current label stipulates that nifurtimox should be taken after meals,^18^ which may help to mitigate gastrointestinal TEAEs. Although this places an additional restriction on an already complex dosing regimen, our studies show that it has a major impact on absorption of the drug based on the striking effect of food on bioavailability. It is known that food can have such effects for a variety of reasons, such as interacting physically or chemically with the drug substance, delaying gastric emptying, stimulating bile release, and changing the pH within the gastrointestinal tract and altering its blood flow.^22^, ^23^ For this reason, the US Food and Drug Administration (FDA) recommends evaluation of bioequivalence following ingestion of a 800‐ to 1000‐kcal meal in which around 50% of the total calories come from fat (as was employed here) to maximize the potential for demonstrating a food effect.^22^

In our analysis, food intake seemed to slow the absorption of nifurtimox slightly based on the change in time from dosing to peak plasma concentration (median t_max_ increased from 3 to 4 hours). Total systemic exposure to nifurtimox was also ≈71% greater after food than in a fasted state. As a poorly soluble drug, prolonged gastric residence of nifurtimox may have contributed to this improvement in oral bioavailablilty. The underlying mechanism for this is unclear, but could relate to enhanced solubility owing to the presence of bile acids, longer residence in the intestine and/or increased splanchnic blood flow after food ingestion.^22^ Nevertheless, systemic exposure in response to a 120‐mg dose of nifurtimox was remarkably consistent across both studies providing that the dose was administered after food.

Historical nifurtimox PK data have been obtained in 2 small studies. One study in 7 healthy volunteers, each receiving a single oral dose of nifurtimox 15 mg/kg, determined the elimination t_½_ to be 2.95 hours and t_max_ to be 2.24 hours.^10^ A subsequent study investigated nifurtimox PK characteristics in 7 patients with chronic renal failure receiving regular hemodialysis.^24^ AUC and C_max_ for nifurtimox, also after a single oral dose of nifurtimox 15 mg/kg, were 70%‐75% higher in patients with renal failure than in the healthy volunteers (AUC, 9510 vs 5430 μg·h/L, respectively; C_max_, 1290 vs 751 μg/L, respectively; values in patients on dialysis were determined on a nondialysis day).^10^, ^24^ No significant differences in t_½_ or t_max_ were seen between the 2 patient groups. These observed differences in exposure and in C_max_ between the studies were similar in magnitude to the differences in these parameters between fed and fasting patients in our studies. As fed/fasting status was not disclosed in the report on patients with renal failure, it may be the case that the greater systemic availability in these patients was caused by a food effect.^24^ Were the differences attributable to poorer drug clearance, a longer elimination half‐life than in healthy individuals might also have been expected in those with renal failure.

The absorption and elimination profiles of nifurtimox in these studies following oral ingestion under fasted conditions were similar to the findings under fasting conditions in the current study in patients with Chagas disease. Although the C_max_ in healthy controls (751 μg/L)^10^ was higher than that observed in plasma here (277 μg/L), the dose employed (15 mg/kg) was considerably higher than that administered in our studies (120 mg total dose or up to 2 mg/kg). As well as differences in dose, methodological differences between the historical studies and the current study generally impede their direct comparison.

Nifurtimox was well tolerated in the current studies, whether administered as a single 120‐mg tablet, as four 30‐mg tablets, or as an aqueous slurry made from four 30‐mg tablets and also when given under fed or fasted conditions; there were no new safety or tolerability findings. The most common TEAEs observed during the studies were gastrointestinal disorders (vomiting, nausea, and abdominal pain) and headache, which are known side effects of nifurtimox.^4^ All nifurtimox‐related TEAEs were mild or moderate in intensity and resolved, mostly within a day, as has previously been reported with the drug.^15^, ^25^, ^26^ Although it is difficult to draw conclusions about safety and tolerability from relatively small, single‐dose studies, the frequency and type of TEAEs were broadly the same across all intervention groups, and no severe or serious events were reported. The overall rate of TEAEs was greater in study A than in study B, but there is no obvious clinical explanation for this difference; it may be attributable to relatively small sample sizes. In terms of drug‐related safety and tolerability, taking nifurtimox after food may reduce the likelihood of gastrointestinal TEAEs compared with taking them when fasting; however, these events were infrequent in our study, so no conclusions can be drawn.

We followed FDA guideline recommendations for the bioequivalence study and took a comparable approach for the food‐effect study. Our studies employed randomized, 2‐period crossover designs with an intervening washout of at least 5 half‐lives (equating to <1 day for nifurtimox, whereas our studies included a washout of ≥5 days). The smallest group in our studies was 12 individuals, the threshold sample size advocated in the guidelines, with twice that number being enrolled in the demonstration of bioequivalence. The test conditions (fasting period, diet, fluid intake, activity levels, time of ingestion, etc.) and sampling conditions all followed guideline recommendations, as did the fat and calorie content of the standard meal. It should be noted that the effect of food on the PK of nifurtimox reported here may be less marked among individuals infected with Chagas disease, who do not typically eat the type of guideline‐recommended high‐fat, high‐calorie meals used in our study. For this reason, a clinical study is underway to investigate the influence on nifurtimox PK of diets that are representative of those among children typically encountered in routine clinical practice (eg, diets rich in dairy products).

After food intake, 4 of the new nifurtimox 30‐mg tablets were found to be bioequivalent to the currently marketed nifurtimox 120‐mg tablet in men and women with chronic Chagas disease. In addition, oral administration as an aqueous slurry was found to be equivalent to ingestion of four 30‐mg nifurtimox tablets, offering the possibility of accurate age‐ and body weight‐based dosing of nifurtimox in a format that is simple to prepare and that is manageable for individuals who may choke on whole tablets, such as infants and very young children. Nifurtimox, given either as a single 120‐mg dose or as four 30‐mg tablets, was well tolerated, and no new safety signals were observed. With 2 dose strengths that permit administration in 15‐mg increments, and the option to administer nifurtimox as a slurry, a complex dosing schedule can be translated into a practical regimen for use irrespective of age group in an outpatient setting.

## Conflicts of Interest

H.S. is an employee of Bayer AG. E.F. has nothing to disclose. F.G.‐B. has received funding from the Agencia Nacional de Ciencia y Tecnica and from Consejo Nacional de Investigaciones en Ciencia y Tecnologia (CONICET). J.N. is an employee of Bayer AG and owns stocks in Bayer AG but receives no income in the form of stocks or stock options. B.W. is CEO and owner of Chrestos Concept GmbH and Co. K.G. has no conflicting interests. G.Y. has nothing to disclose. J.A. has received remuneration for consultancy from Bayer. Medical writing support was provided by Highfield, Oxford, United Kingdom and was funded by Bayer AG, Germany.

## Funding

This study was funded and supported by Bayer AG, Germany.

## Author Contributions

H.S. planned and designed the studies, including the study protocols, PK analysis of the data and generated the clinical study reports, as part of the program of clinical development of nifurtimox for the treatment of pediatric patients. As primary author, H.S.’s contributions to this article included the initial concept, critical review, and interpretation and discussion of the biopharmaceutical relevance of the PK findings for patients’ treatment. E.F. was a clinical investigator in the study and reviewed the article critically for publication. F.G.‐B. participated in patient recruitment and critical review and preparation of the article. J.N. supported planning of safety measurements described in study protocols and used in the studies, writing and review of safety‐related parts of clinical study reports, as well as critical review of the article during its preparation. B.W. planned and conducted the statistical analyses and reviewed the article critically at all stages of development. G.Y. was a clinical investigator in the study and reviewed the article critically for publication. J.A. made substantial contributions to the conception and design of the study, was involved in the acquisition and interpretation of data, and reviewed the article critically for publication. All authors approved publication of the article and agree to be accountable for all aspects of the work therein.

## Data‐Sharing Statement

Subject to a confidentiality agreement, the study protocols and reports summarized in this publication can be obtained from Bayer AG, via the corresponding author.

## Supporting information

Supplementary information

Supplementary information

Supplementary information

Supplementary information

Supplementary information

Supplementary information

Supplementary information

Supplementary information
