# Supplementary material for: Biopharmaceutical Characteristics of Nifurtimox Tablets for Age‐ and Body Weight‐Adjusted Dosing in Patients With Chagas Disease
Source: Clin Pharmacol Drug Dev. 2020 Oct 8;10(5):542–55. doi: 10.1002/cpdd.871 (PMC8246722; doi:10.1002/cpdd.871)
Supplement: Supplementary file 1 — Supplementary information [file CPDD-10-542-s001.docx]

**Supplementary Figure Legends**

## Figure S1. Nifurtimox plasma concentrations for participants in Study A. (A) Group 1: 4 x 30 mg tablets or 4 x 30 mg aqueous slurry under fed conditions (N = 12), (B) Group 2: 4 x 30 mg tablets or 1 x 120 mg tablet under fed conditions (N = 24). Data are geometric mean and standard deviation; curves are plotted on a semi-logarithmic scale. LLOQ = lower limit of quantitation.

## Figure S2. Nifurtimox plasma concentrations for participants in Study B receiving 4 x 30 mg tablets in the fasted or fed states (N = 35). Data are geometric mean and standard deviation; LLOQ = lower limit of quantitation.
