# Supplementary figures and images for: Biopharmaceutical Characteristics of Nifurtimox Tablets for Age‐ and Body Weight‐Adjusted Dosing in Patients With Chagas Disease
Source: Clin Pharmacol Drug Dev. 2020 Oct 8;10(5):542–55. doi: 10.1002/cpdd.871 (PMC8246722; doi:10.1002/cpdd.871)

Figure S1a

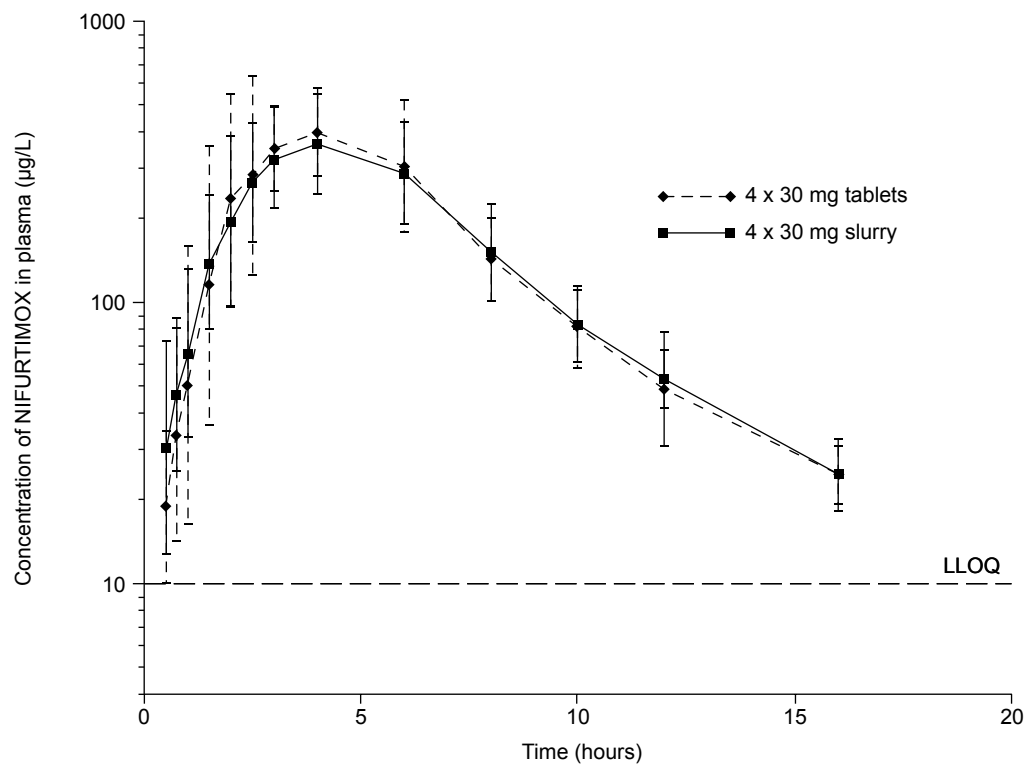

Supplement: Supplementary file 2 — Supplementary information [file CPDD-10-542-s007.pdf]

Figure S1b

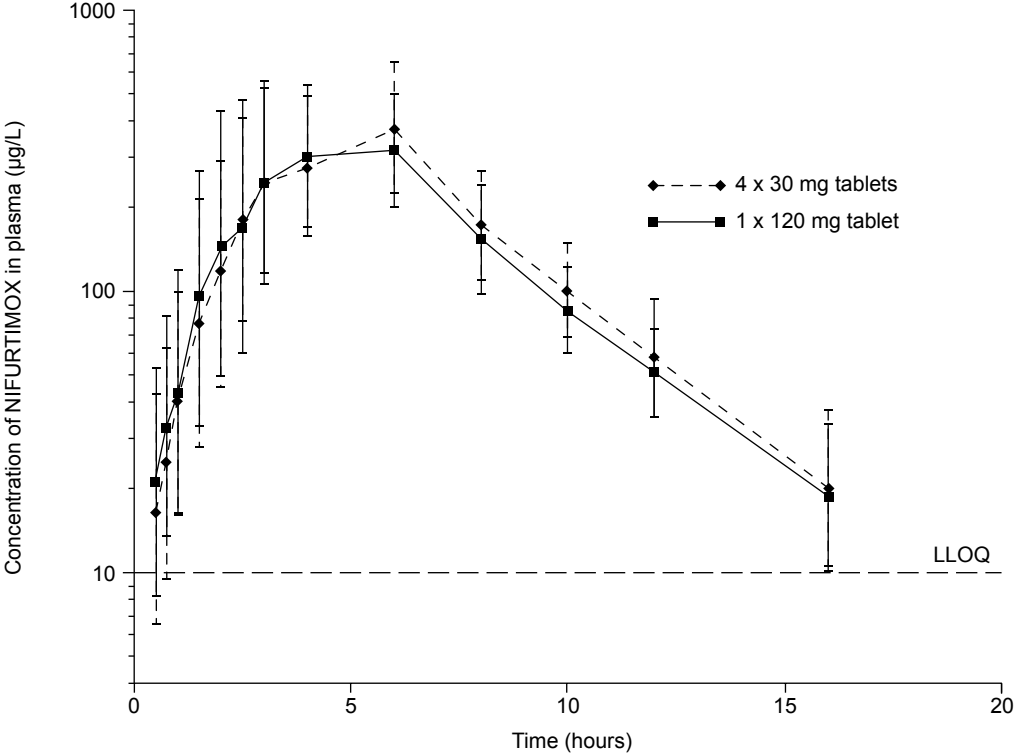

Supplement: Supplementary file 3 — Supplementary information [file CPDD-10-542-s004.pdf]

Figure S2

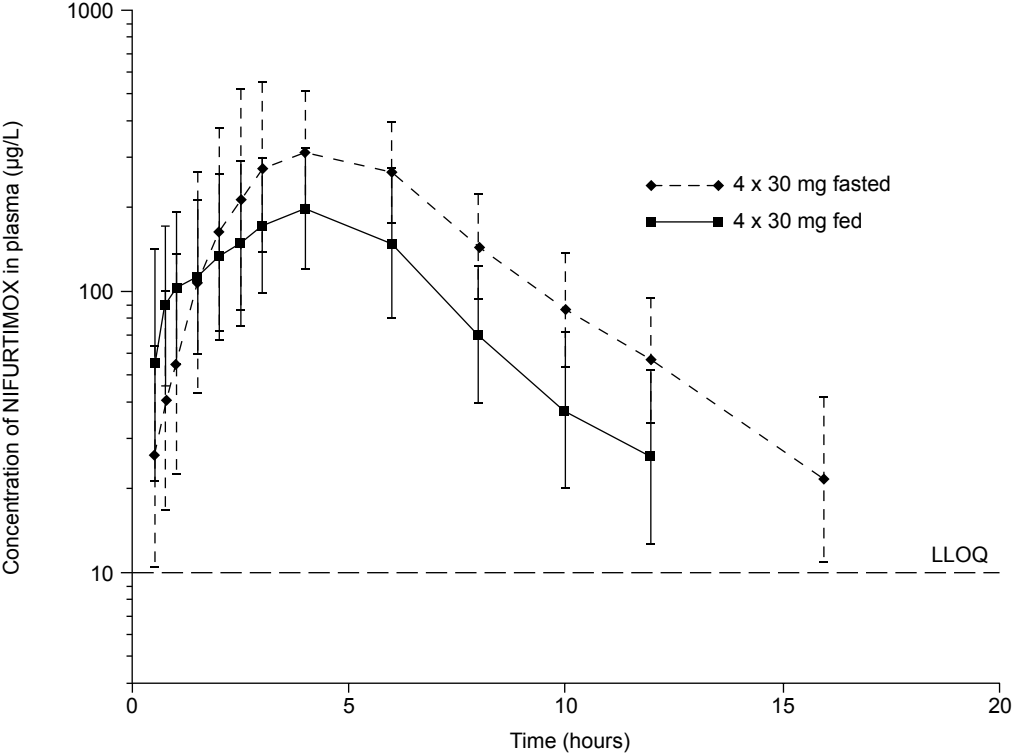

Supplement: Supplementary file 4 — Supplementary information [file CPDD-10-542-s002.pdf]
