# Supplementary material for: Biopharmaceutical Characteristics of Nifurtimox Tablets for Age‐ and Body Weight‐Adjusted Dosing in Patients With Chagas Disease
Source: Clin Pharmacol Drug Dev. 2020 Oct 8;10(5):542–55. doi: 10.1002/cpdd.871 (PMC8246722; doi:10.1002/cpdd.871)
Supplement: Supplementary file 5 — Supplementary information [file CPDD-10-542-s003.docx]

**Table S1. Inclusion criteria (Study A and Study B).**

| Diagnosis of chronic Chagas disease: previous diagnosis of acute or chronic Chagas disease by a health clinic prior to study screening. Diagnosis of chronic Chagas disease could be made by clinical findings supported by antibody titers, if available. |
| --- |
| Aged 18-45 years |
| Body mass index ≥18 and <29.9 kg/m^2^ |
| Women of childbearing potential with confirmed last menstrual period and a negative pregnancy test at screening or women of non-childbearing potential (e.g. surgically sterile). |
| Participants of reproductive potential agreed to use two reliable forms of contraception when sexually active during the study and for 12 weeks after the last dose of study medication. |
| Male subjects agreed not to act as sperm donor for a period of 12 weeks after dosing. |
| Women of childbearing potential with confirmed last menstrual period and a negative pregnancy test at screening or women of non-childbearing potential (e.g. surgically sterile). |
| Participants of reproductive potential agreed to use two reliable forms of contraception when sexually active during the study and for 12 weeks after the last dose of study medication. |
| At least 3 months since delivery or abortion, or 3 months since cessation of lactation before the first screening visit |
