# Supplementary material for: Biopharmaceutical Characteristics of Nifurtimox Tablets for Age‐ and Body Weight‐Adjusted Dosing in Patients With Chagas Disease
Source: Clin Pharmacol Drug Dev. 2020 Oct 8;10(5):542–55. doi: 10.1002/cpdd.871 (PMC8246722; doi:10.1002/cpdd.871)
Supplement: Supplementary file 6 — Supplementary information [file CPDD-10-542-s008.docx]

**Table S2. Exclusion criteria (Study A and Study B).**

| **Medical and surgical history** |
| --- |
| Acute Chagas disease |
| Known sensitivity to the study drug |
| Incompletely cured pre-existing diseases (except chronic Chagas disease without active gastrointestinal condition) |
| Clinically significant allergies, non-allergic drug reactions, or multiple severe drug allergies |
| Unstable or uncontrolled medical conditions |
| Febrile illness <1 week before the first study drug administration |
| **Medication, drug use and special behavioral patterns** |
| Use of medicines, prescribed or over the counter, or use of recreational drugs |
| Smoking within 3 months prior to study drug administration and throughout the study |
| Use of systemic or topical medicines or substances which oppose the study objective or may influence them within 4 weeks prior to the first study drug administration |
| Regular consumption of > 20 mg alcohol/day or current or history of drug or alcohol abuse within 6 months of screening |
| Special diets preventing subjects from eating standard meals during the study |
| Regular consumption of >2 L of xanthine-containing beverages |
| Intake of foods or beverages containing grapefruit within 2 weeks before the first study drug administration |
| Donation of blood or plasmapheresis within 8 weeks before the first study drug administration |
| Donation of more than 100 mL of blood within 4 weeks or 500 mL within 3 months before the first study drug administration |
| **Electrocardiogram, blood pressure, heart rate** |
| Clinically relevant findings in the electrocardiogram such as a second- or third-degree atrioventricular block, or prolongation of the QRS complex over 120 msec or of the QT interval corrected according to Bazett’s formula over 450 msec |
| Systolic blood pressure <100 or >140 mmHg or diastolic blood pressure <50 or >90 mmHg (after at least 15 min sitting) |
| Heart rate <45 or >95 beats/min (after at least 15 min sitting) |
| **Physical and laboratory examination** |
| Findings that would have excluded the subject in the physician’s judgment e.g., enlarged liver, irregular heartbeat, undiagnosed acute illness, melanoma |
| Positive pregnancy test |
| Positive results for hepatitis B virus surface antigen, hepatitis C virus antibodies human immunodeficiency virus antibodies |
| Positive urine drug screening |
| Clinically relevant deviations of the screened laboratory parameters from reference ranges |
