# Supplementary material for: Biopharmaceutical Characteristics of Nifurtimox Tablets for Age‐ and Body Weight‐Adjusted Dosing in Patients With Chagas Disease
Source: Clin Pharmacol Drug Dev. 2020 Oct 8;10(5):542–55. doi: 10.1002/cpdd.871 (PMC8246722; doi:10.1002/cpdd.871)
Supplement: Supplementary file 7 — Supplementary information [file CPDD-10-542-s006.docx]

**Table S3.** Pharmacokinetic parameters of nifurtimox in Study A (geometric mean [% CV], PK analysis set)^a^

|  | **Group 1** | | **Group 2** | |
| --- | --- | --- | --- | --- |
| **Parameter** | **Intervention A 4 x 30 mg tablets (N=12)** | **Intervention B 4 x 30 mg aqueous slurry (N=12)** | **Intervention A 4 x 30 mg tablets (N=24)** | **Intervention C 1 x 120 mg tablet (N=24)** |
| AUC, μg.h/L | 2790 (18.8) | 2630 (21.8) | 2670 (25.8) | 2550 (25.0) |
| AUC (0–t_last_), μg.h/L | 2670 (19.2) | 2490 (23.5) | 2560 (26.3) | 2450 (25.6) |
| AUC_norm_, μg.h/L | 1490 (15.3) | 1410 (22.9) | 1390 (19.8) | 1330 (17.7) |
| C_max_, μg/L | 568 (26.4) | 434 (31.2) | 518 (40.3) | 509 (37.9) |
| C_max,norm_, kg/L | 304 (18.8) | 233 (26.8) | 270 (37.7) | 266 (37.0) |
| t_max_, h^a^ | 4.0 (2.0–6.0) | 4.0 (2.0–8.0) | 4.0 (2.0–6.0) | 4.0 (2.0–6.0) |
| t_½_, h | 3.30 (11.6) | 3.61 (37.3) | 2.63 (23.1) | 2.85 (30.2) |

AUC, area under the plasma concentration curve; AUC (0–t_last_), AUC from baseline to last measurable concentration; AUC_norm_, AUC from baseline to infinity normalized for dose/kg bodyweight; C_max_, maximum observed concentration; C_max,norm_, C_max_ normalized to dose and body weight; CV, coefficient of variation; PK, pharmacokinetic; t_max_, time to reach C_max_; t_½_, half-life;. All dosing was under fed conditions; SD, standard deviation. ^a^Median (range)
