# Supplementary material for: Biopharmaceutical Characteristics of Nifurtimox Tablets for Age‐ and Body Weight‐Adjusted Dosing in Patients With Chagas Disease
Source: Clin Pharmacol Drug Dev. 2020 Oct 8;10(5):542–55. doi: 10.1002/cpdd.871 (PMC8246722; doi:10.1002/cpdd.871)
Supplement: Supplementary file 8 — Supplementary information [file CPDD-10-542-s005.docx]

## **Table S4.** Pharmacokinetic parameters of nifurtimox in Study B (geometric mean [% CV]; PK analysis set)^a^

| **Parameter** | **Intervention D 4 x 30 mg tablets, fasted (N=35)** | **Intervention E**  **4 x 30 mg tablets, fed (N=35)** |
| --- | --- | --- |
| AUC, μg.h/L | 1480 (40.4) | 2530 (21.3) |
| AUC (0–t_last_), μg.h/L | 1390 (40.6) | 2400 (21.7) |
| CL/F, L/h | 81.2 (40.4) | 47.4 (21.3) |
| C_max_, μg/L | 277 (36.8) | 465 (33.4) |
| t_max_, h^a^ | 3.0 (0.5–6.1) | 4.0 (1.0–8.0) |
| t_½_, h | 3.07 (34.6) | 3.13 (27.4) |

AUC, area under the concentration curve; AUC (0–t_last_), AUC from baseline to last measurable concentration; CL/F, apparent total body clearance; C_max_, maximum observed concentration; CV, coefficient of variation; PK, pharmacokinetic; t_max_, time to reach C_max_; t_½_, half-life. ^a^Median (range)
